# Durable protection against repeated penile exposures to simian-human immunodeficiency virus by broadly neutralizing antibodies

**DOI:** 10.1038/s41467-020-16928-9

**Published:** 2020-06-24

**Authors:** David A. Garber, Debra R. Adams, Patricia Guenthner, James Mitchell, Kristen Kelley, Till Schoofs, Anna Gazumyan, Martha Nason, Michael S. Seaman, Janet McNicholl, Michel C. Nussenzweig, Walid Heneine

**Affiliations:** 1 Laboratory Branch, Division of HIV/AIDS Prevention, Centers for Disease Control and Prevention, Atlanta, GA USA; 2 Laboratory of Molecular Immunology, The Rockefeller University, New York, NY USA; 3 Biostatistics Research Branch, National Institute of Allergy and Infectious Diseases, National Institutes of Health, Rockville, MD USA; 4 Center for Virology and Vaccine Research, Beth Israel Deaconess Medical Center, Harvard Medical School, Boston, MA USA; 5 Howard Hughes Medical Institute, The Rockefeller University, New York, NY USA; 6 Present Address: GSK Vaccines, 1300 Wavre, Belgium

**Keywords:** Vaccines, Virology

## Abstract

Penile acquisition of HIV accounts for most infections among men globally. Nevertheless, candidate HIV interventions for men advance to clinical trials without preclinical efficacy data, due primarily to a paucity of relevant animal models of penile HIV infection. Using our recently developed macaque model, we show that a single subcutaneous administration of broadly neutralizing antibody (bNAb) 10-1074 conferred durable protection against repeated penile exposures to simian-human immunodeficiency virus (SHIV_SF162P3_). Macaques co-administered bNAbs 10-1074 and 3BNC117, or 3BNC117 alone, also exhibited significant protection against repeated vaginal SHIV_AD8-EO_ exposures. Regression modeling estimated that individual plasma bNAb concentrations of 5 μg ml^−1^ correlated with ≥99.9% relative reduction in SHIV infection probability via penile (10-1074) or vaginal (10-1074 or 3BNC117) challenge routes. These results demonstrate that comparably large reductions in penile and vaginal SHIV infection risk among macaques were achieved at clinically relevant plasma bNAb concentrations and inform dose selection for the development of bNAbs as long-acting pre-exposure prophylaxis candidates for use by men and women.

## Abstract (web summary)

Here, using a repeat-exposure penile SHIV infection model in macaques, the authors show that a single subcutaneous administration of a broadly neutralizing antibody (bNAb) exerts long-acting protection against both penile and vaginal virus exposure, which highlights bNAbs as a potential therapeutic for LA-PrEP.

## Introduction

HIV infection via the penis accounts for a majority of infections among the 17 million men estimated to be living with HIV globally, yet remains a relatively understudied route of virus transmission. It is the primary route of HIV acquisition among heterosexual men and is of relevance to MSM who practice insertive anal intercourse^1^. Current strategies to reduce one’s risk of acquiring HIV infection via the penis include condom usage, male circumcision and taking antiretroviral drugs for preexposure prophylaxis (PrEP)^2–5^. Regrettably, each of these prevention strategies has limitations that curb effectiveness. For example, even when used consistently, condoms are <80% effective in reducing HIV incidence among uninfected partners of HIV-discordant heterosexual couples^2^. Similarly, male circumcision, in which all or part of the penile foreskin is removed surgically, confers incomplete protection against HIV–reducing infection risk by 50–60%^5^. With regard to PrEP, once-daily oral tenofovir disoproxil fumarate (TDF) or TDF-emtricitabine (FTC) combination reduced HIV infection risk by 63% or 84%, respectively, among heterosexual men with a known HIV-infected partner^3^. Among MSM, clinical trials have shown daily oral TDF–FTC to reduce overall HIV incidence either by 44% (with significantly greater reductions (92–95%) among trial participants who had detectable study drugs in plasma)^6^, or by 86% (PROUD Study)^7^. In addition, on-demand dosing with TDF–FTC, rather than daily dosing, demonstrated an 86% reduction in HIV incidence as compared with placebo controls (IPERGAY Study)^8^. While oral TDF–FTC can be highly efficacious in preventing HIV infection, users’ lack of adherence to drug regimens is a major behavioral impediment that limits PrEP effectiveness^9^.

As a result, development of long-acting HIV intervention products that require relatively less frequent dosing remains a top priority in the HIV prevention and treatment fields. Passive immunization using recombinant monoclonal antibodies capable of neutralizing diverse HIV isolates (broadly neutralizing antibodies (bNAbs)) is a promising approach to achieve long-acting HIV prevention. The Antibody-Mediated Prevention (AMP) Study currently is evaluating bNAb VRC01, when administered via intravenous infusion once every 8 weeks, for safety and efficacy to prevent HIV infection among high-risk women (HVTN 703/HPTN 081; NCT02568215) and among men and transgender persons who have sex with men (HVTN 704/HPTN 085; NCT02716675)^10^. However, because HIV transmission among MSM occurs more commonly via the rectal than the penile route^1,11^, the AMP Study is not poised specifically to evaluate antibody-mediated protection against penile HIV acquisition.

Many other bNAbs, which exhibit greater potency or neutralization breadth than VRC01 are being developed and are in clinical evaluation^12–14^. Some of the most clinically advanced among these are 10–1074, which targets the base of the third variable loop and surrounding glycans on the HIV envelope protein (Env), and 3BNC117, which targets the CD4 binding site^15,16^. Both have been shown to be safe and well tolerated in HIV-infected and uninfected individuals, and capable of suppressing HIV viremia in viremic subjects^12,17–20^.

During preclinical development, HIV prevention products or vaccines often are evaluated for protective efficacy in nonhuman primate models. These infection models are predicated upon exposing macaques to a single high-dose, or repeated low-doses of simian-human immunodeficiency virus (SHIV), most commonly via rectal or vaginal routes^21,22^. bNAbs 10–1074 and 3BNC117 have been shown to protect macaques against rectal infection with SHIV_AD8-EO_^23,24^. However, their efficacy in preventing infection via the other major mucosal routes of HIV infection—namely penile or vaginal, has not been described. Nor is it known how these bNAbs’ correlates of protection compare among all mucosal infection routes.

In the absence of penile animal model data, it is difficult to determine how efficacious PrEP regimens are in preventing HIV infection across the penile mucosa during insertive intercourse (vaginal or rectal) with an infected individual. Myriad differences exist between penile, vaginal, and rectal mucosa that may modulate HIV susceptibility or PrEP pharmacokinetics. These include differences in epithelial thickness or composition, presence or absence of mucus, mechanical trauma incurred via sexual intercourse, or prevalent microbiota^25–32^. Despite the public health relevance of penile HIV infection, preclinical studies of the efficacy of PrEP or other biomedical preventions against this route are limited, due largely to a lack of relevant animal models. This represents a knowledge gap with unknown impact on current and future translational efforts to develop new candidate HIV interventions for men.

Recently, we developed a repeat-exposure penile SHIV infection model in rhesus macaques to evaluate the protective efficacy of biomedical preventions against HIV^33^. The model is predicated upon repeatedly exposing multiple penile tissues that are relevant for HIV acquisition in humans, including the inner foreskin, glans, and distal urethra, to limiting doses of SHIV_SF162P3_ (i.e., doses that do not result in systemic infection among all animals following a single challenge) using a nontraumatic technique that precludes inadvertent perturbation of the penile epithelium^33^. Here, we utilized this penile infection model to assess the protection conferred by passive bNAb immunization. We chose to evaluate 10–1074, which exhibits potent neutralization of SHIV_SF162P3_, and show that a single subcutaneous dose of 10–1074 durably protected macaques against repeated penile SHIV_SF162P3_ challenges.

Because the efficacy of 10–1074 against vaginal challenge had not been evaluated, we also tested its protective efficacy in a repeat low dose challenge model. However, the greatest burden of HIV infection of women is in sub-Saharan Africa, where multiple HIV-1 subtypes predominate^34^. In vivo, in vitro, and in silico modeling predicts that bNAbs used in combination will achieve greater neutralization coverage^35–40^. To this end, several phase-I studies evaluating bNAb combinations are ongoing^14^, and one that evaluated administering 10–1074 in combination with 3BNC117 recently has been reported^20^. Therefore, we elected to evaluate 10–1074 in combination with 3BNC117. Because SHIV_SF162P3_ is not sensitive to neutralization by 3BNC117, we chose to challenge vaginally with SHIV_AD8-EO_, which is sensitive to both 3BNC117 and 10–1074. As such, our penile and vaginal challenge models used SHIVs that are similarly susceptible to neutralization by 10–1074, with IC_80_ values of 0.20 or 0.25 μg ml^−1^ against the replication competent challenge virus stock of SHIV_SF162P3_ or SHIV_AD8-EO_, respectively. We show that subcutaneously delivered 10–1074 and 3BNC117 exhibited different pharmacokinetics including a longer plasma persistence of 10–1074 than 3BNC117, providing an opportunity to measure efficacy against vaginal challenge of the bNAb combination and of 10–1074 alone. We used probit regression analyses to compare plasma bNAb concentrations that reduced penile or vaginal infection.

## Results

### Protection by 10–1074 against repeated penile SHIV challenges

**Fig. 1:**
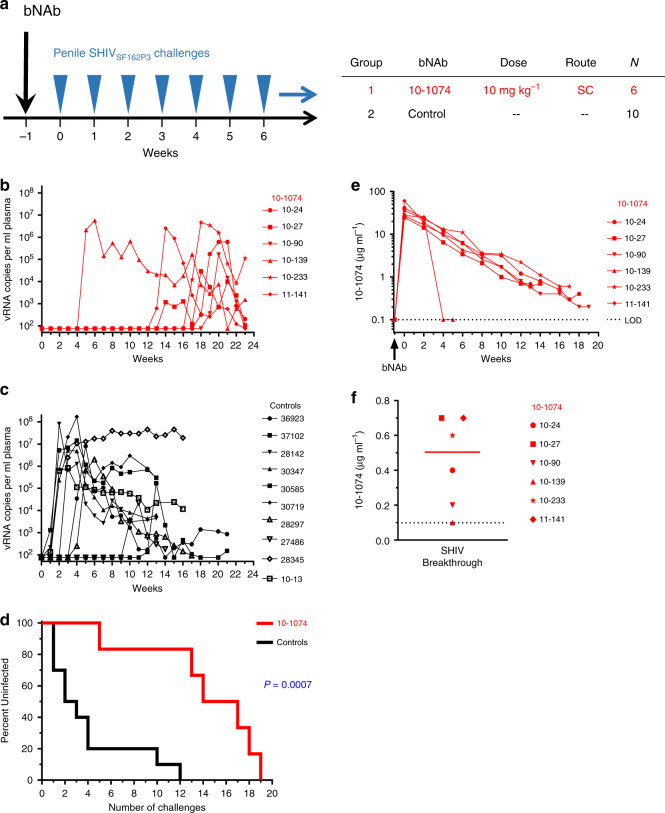
Passive immunization of macaques with bNAb 10–1074 delays SHIV acquisition following repeated penile SHIV challenges. **a** Study design to assess the protective efficacy of 10–1074 against repeated penile SHIV challenges. Rhesus macaques (*N* = 6; Indian origin) were injected subcutaneously once with 10–1074 (10 mg kg^−1^). Beginning 1 week later, macaques were challenged repeatedly, once per week, via the penis with SHIV_SF162P3_ (200 TCID_50_ into the prepuce pouch and 16 TCID_50_ into the distal urethra) until systemic SHIV infection was confirmed via plasma viral load assay. Control macaques (*N* = 10) received no antibody, but were challenged identically. Plasma viral load (vRNA copies ml^−1^) determined in 10–1074-treated macaques (**b**) or untreated controls (**c**). Symbols denote individual animals. **d** Percentages of macaques remaining uninfected at 7 days following administration of the indicated cumulative number of SHIV challenges. Statistical difference between groups was measured using a two-sided log-rank test (*P* = 0.0007). **e** Plasma levels of 10–1074 (μg ml^−1^) were determined via TZM-bl neutralization assays using 10–1074-sensitive pseudovirus X2088.c9. **f** Plasma concentrations of 10–1074 in individual macaques (*N* = 6) at the time of SHIV breakthrough (values from **e**).The solid line denotes the group median; the dotted line denotes the limit of detection for 10–1074. Source data are provided in Supplementary Tables or a Source data file. SHIV infection data for animals in the control group have been reported previously in a paper describing the development of our penile SHIV infection model^33^ and are reproduced here to facilitate comparison between the treatment and control groups.

We sought to determine the protective efficacy that passive immunization with 10–1074 conferred against SHIV acquisition by the penile route in rhesus macaques. Six macaques in the treatment group (Group-1) received a single subcutaneous injection of 10–1074 (human IgG_1_ isotype; does not contain the LS or any other half-life modifying mutations) at 10 mg per kg body weight at 1 week prior to the start of SHIV challenges (Fig. 1a). We performed nontraumatic penile challenges with SHIV using a ‘no contact’ technique that eliminated inadvertent perturbation of the penile mucosa during the inoculation procedure itself, while simultaneously exposing multiple susceptible penile tissues to the challenge virus^33^. For each virus challenge, macaques received 200 TCID_50_ SHIV_SF162P3_ into the prepuce pouch and 16 TCID_50_ SHIV_SF162P3_ into the distal urethra. Ten historical control animals (Group-2) had received no antibodies and had been challenged identically with the same stock of SHIV_SF162P3_ (Fig. 1a). Penile SHIV_SF162P3_ challenges were repeated once weekly until systemic infections were confirmed via positive RT-qPCR plasma viral load assay for macaques in the treatment group (Fig. 1b) or control group (Fig. 1c). Macaques that received a single injection of 10–1074 became infected following a median of 15.5 weekly challenges (range = 5–19) and were protected significantly longer than untreated control animals, which became infected following a median of 2.5 challenges (range = 1–12) (*P* = 0.0007, Log-Rank test) (Fig. 1d). Of note, the earliest infection among macaques administered 10–1074 occurred in animal 10–139 following five SHIV_SF162P3_ challenges and corresponded with an abrupt decline in the neutralization activity of 10–1074 in plasma between 3 and 5 weeks post injection, due to the development of an antidrug antibody (ADA) response (Fig. 1b, Supplementary Fig. 1)^41,42^. Protection among 10–1074-treated macaques that did not exhibit such ADA responses was relatively greater, showing median protection against 17 weekly challenges (range = 13–19). No differences in levels of peak viremia, or area under the curve (AUC) through 7 weeks post infection were observed between 10–1074-treated and control groups (Supplementary Fig. 1).

Plasma levels of 10–1074 exhibited a mean maximum concentration (*C*_max_) of 36.0 ± 13.3 μg ml^−1^ at 7 days following antibody injection, which was the earliest postinjection timepoint assayed (Fig. 1e). Plasma levels of 10–1074 exhibited an average half-life of 15.5 ± 4.0 days and remained above the level of detection (0.10 μg ml^−1^) for 15–20 weeks following injection in 5 of 6 treated animals (Fig. 1e; (Supplementary Table 1)). At the time of SHIV breakthrough (i.e., first detectable plasma viremia), the median plasma concentration of 10–1074 among all treated macaques was 0.50 μg ml^−1^ (range: <0.10–0.70 μg ml^−1^) (Fig. 1f). Excluding the animal that developed an early ADA response against 10–1074 and had an undetectable level of 10–1074 at the time of SHIV breakthrough, the median plasma concentration of 10–1074 at the time of SHIV breakthrough was 0.60 μg ml^−1^ (range of 0.20–0.70 μg ml^−1^).

**Fig. 2:**
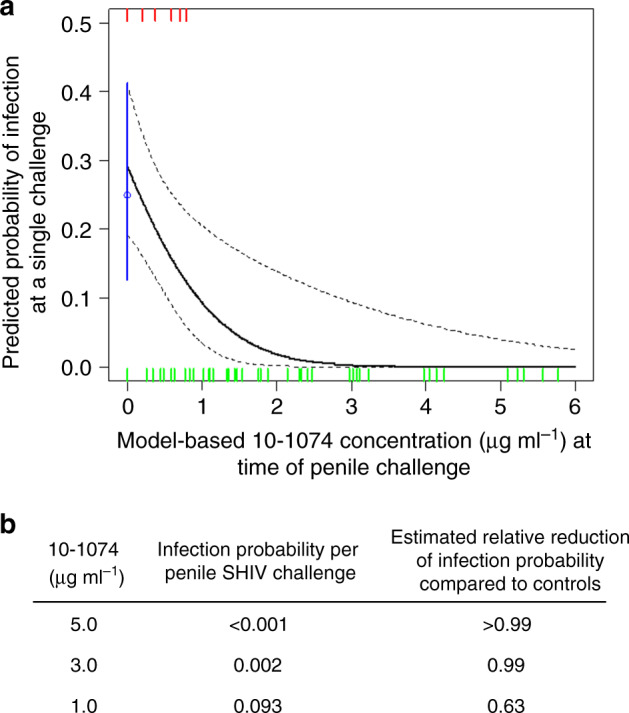
10–1074 antibody concentration predicts the probability of infection following penile SHIV_SF162P3_ challenge. **a** Probit regression analysis was used to estimate the per-challenge probability of penile SHIV acquisition as a function of imputed 10–1074 concentration in plasma. The per-challenge probability of infection among untreated control animals was 0.25 (95% CI 0.13–0.41) and is denoted in blue. Tick marks represent SHIV challenge events that either did (red) or did not (green) result in systemic infection. The solid black line is the model-based prediction; dotted black lines depict 90% pointwise confidence intervals. **b** Model-based estimates of SHIV infection probabilities following a single penile challenge and reduction of infection probability as compared with controls, at the indicated plasma concentrations of 10–1074.

To evaluate antibody-mediated protection against penile infection, we used probit regression to estimate the per-challenge probability of SHIV infection as a function of imputed plasma 10–1074 levels (Fig. 2), excluding animal 10–139 who exhibited an early ADA response. Among no-antibody controls, each SHIV challenge presented a 0.25 chance of infection (Fig. 2; 95% CI 0.13–0.41). The probit model estimated >100-, 9.5-, and 1.6-fold reductions of this infection probability to <0.001, 0.002, or 0.093 for plasma 10–1074 concentrations of 5.0, 3.0, or 1.0 μg ml^−1^, respectively. Bootstrap fitting of the probit regression model estimated a 0.01 probability of infection following a single penile SHIV challenge at a plasma 10–1074 concentration of 2.28 μg ml^−1^ (95% CI: 1.27, 3.47 μg ml^−1^).

### Protection by bNAbs against repeated vaginal SHIV challenges

bNAbs 10–1074 and 3BNC117 are among the most clinically advanced second generation bNAbs and have not been evaluated previously in nonhuman primate models for protection against vaginal SHIV challenge. Because bNAbs targeting nonoverlapping epitopes on HIV Env could be used in combination to achieve additive neutralization activity or increased breadth of neutralization^35–40^, we evaluated protection against repeated vaginal SHIV challenges among macaques that received a combination of 10–1074 and 3BNC117, or 3BNC117 singly.

**Fig. 3:**
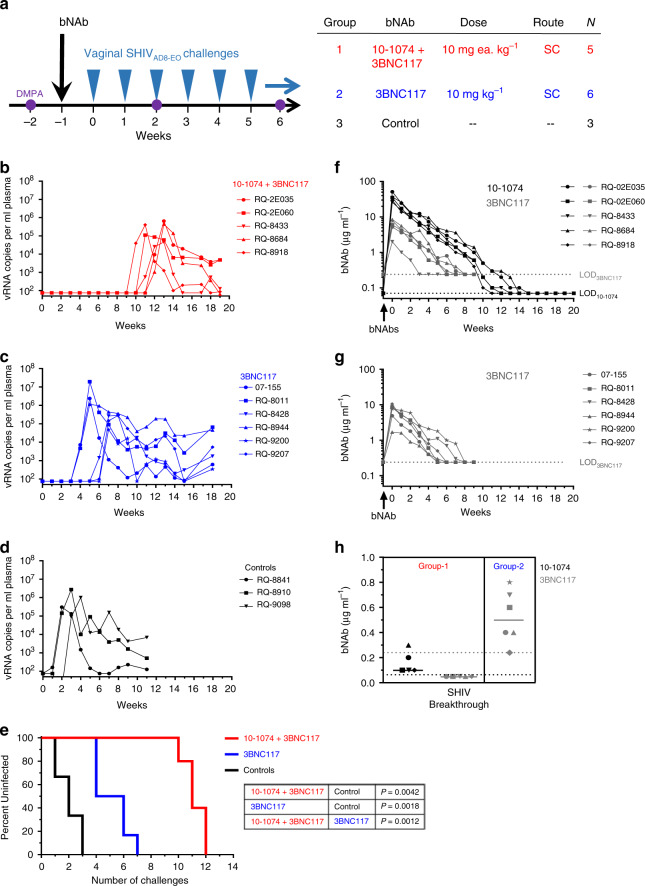
Passive immunization with bNAbs 10–1074 and 3BNC117 in combination, or 3BNC117 singly, delays SHIV acquisition following repeated vaginal challenges in DMPA-treated macaques. **a** Rhesus macaques (Chinese origin) were injected subcutaneously once with a combination of bNAbs 10–1074 and 3BNC117 (10 mg each kg^−1^; *N* = 5) or 3BNC117 singly (10 mg kg^−1^; *N* = 6). Commencing 1 week later, macaques were challenged repeatedly, once per week, intravaginally with SHIV_AD8-EO_ (300 TCID_50_) until systemic SHIV infection was confirmed via plasma viral load assay. Control macaques (*N* = 3) received no antibody but were challenged identically. Animals in both groups received DMPA (30 mg) intramuscularly at 2 weeks before the first SHIV challenge and every 4 weeks thereafter to normalize SHIV susceptibility. Plasma viral loads (vRNA copies ml^−1^) for macaques in the combination bNAb group (**b**), single bNAb group (**c**) or untreated control group (**d**). Symbols denote individual animals. **e** Percentages of macaques remaining uninfected at 7 days following administration of the indicated cumulative number of SHIV challenges. Statistical differences between groups were analyzed using a two-sided log-rank test. Plasma levels of 10–1074 (μg ml^−1^) or 3BNC117 (μg ml^−1^) were determined via TZM-bl neutralization assays using 10–1074-sensitive pseudovirus X2088.c9 or 3BNC117-sensitive pseudovirus Q769.d22 for macaques that received both 10–1074 and 3BNC117 (**f**) or 3BNC117 alone (**g**). **h** Plasma concentrations of 10–1074 or 3BNC117 in individual macaques (*N* = 5 in Group-1, *N* = 6 in Group-2) at the time of SHIV breakthrough (values from **f** (Group-1) or **g** (Group-2)). Solid lines denote group medians; dotted lines indicate the limits of detection for 10–1074 (black) or 3BNC117 (gray). Source data are provided in Supplementary Tables or a Source data file.

Macaques in the treatment groups (Group-1, -2) received a one-time subcutaneous injection of both 10–1074 and 3BNC117 (10 mg each kg^−1^, Group-1) or 3BNC117 singly (10 mg kg^−1^, Group-2) 1 week prior to the start of vaginal SHIV_AD8-EO_ challenges. Both bNAbs were of human IgG_1_ isotype and did not contain the LS or any other half-life modifying mutations. Macaques in the control group (Group-3) were not administered any antibodies and were challenged identically with SHIV_AD8-EO_ (Fig. 3a). Macaques in all groups received intramuscular injections of depot medroxyprogesterone acetate (DMPA; 30 mg) 2 weeks prior to the first SHIV challenge (corresponding to 1 week prior to bNAb administration in the treatment groups), and every 4 weeks thereafter, to normalize vaginal SHIV susceptibility via progestin-mediated thinning of the vaginal mucosa^43–45^ (Fig. 3a). Vaginal SHIV_AD8-EO_ challenges (300 TCID_50_) were repeated once weekly until systemic infections were confirmed via positive RT-qPCR viral load assay for macaques in the combination treatment group (Fig. 3b), 3BNC117-only group (Fig. 3c), or untreated control group (Fig. 3d).

Macaques that received a single injection of only 3BNC117 showed breakthrough SHIV infections following a median of five challenges (range = 4–7) and were protected significantly longer than were untreated controls, which became infected following a median of two SHIV challenges (range = 1–3) (*P* = 0.0018, Log-Rank test; Fig. 3e). More importantly, macaques in the 10–1074 + 3BNC117 combination group were protected against a median of 11 weekly challenges (range = 10–12), which was significantly greater than that conferred following administration of 3BNC117 alone (*P* = 0.0012, Log-rank test) or observed among untreated controls (*P* = 0.0042, Log-Rank test) (Fig. 3e). Levels of peak viremia and early SHIV replication, measured as vRNA AUC through 7 weeks post infection, were similar among all groups (Supplementary Fig. 3).

Beginning 1 week following antibody injection, plasma levels of 10–1074 and 3BNC117 were measured among macaques that had received these two antibodies in combination. The maximum concentration of 10–1074 (mean *C*_max_ 10–1074 = 35.7 ± 9.6 μg ml^−1^) was 6 times as high as 3BNC117 (mean *C*_max_ 3BNC117 = 5.9 ± 2.6 μg ml^−1^) (*P* = 0.0022, paired *t*-test; Fig. 3f), and both maxima were observed at the earliest timepoint (7 days) that was sampled following antibody injection. Among macaques that received only 3BNC117, the mean maximum plasma level of 3BNC117 (mean *C*_max_^3BNC117^ = 7.0 ± 3.2 μg ml^−1^; Fig. 3g) also was observed at the earliest timepoint sampled following antibody injection (7 days post injection) and was similar in magnitude to that observed for 3BNC117 among animals in the combination treatment group (Fig. 3f; Supplementary Tables 2, 3). Thus, no adverse interaction by 10–1074 on 3BNC117 pharmacokinetics was observed in vivo.

In macaques that were administered 3BNC117 singly, the median plasma 3BNC117 concentration at the time of SHIV breakthrough was 0.50 μg ml^−1^ (range = 0.24–0.80 μg ml^−1^) (Fig. 3h). In contrast, at the time of SHIV breakthrough, macaques that had been administered both bNAbs exhibited a median plasma concentration for 10–1074 of 0.10 μg ml^−1^ (range = 0.10–0.30 μg ml^−1^), but undetectable levels of 3BNC117 (Fig. 3h). Moreover, among all macaques in the combination bNAb group, 3BNC117 levels were at, or had been below, the level of detection (0.24 μg ml^−1^) for a median of 4 weeks (range = 3–8 weeks) prior to SHIV breakthrough. This argues that the more durable protection observed in the combination antibody group was due to 10–1074, which persisted relatively longer than 3BNC117. As such, these macaques were protected through a period of effective 10–1074 monotherapy. This allowed us to estimate levels of 10–1074 that were protective against vaginal SHIV_AD8-EO_ infection, and enabled a comparison of correlates of protection for 10–1074 between the penile and vaginal challenge models.

**Fig. 4:**
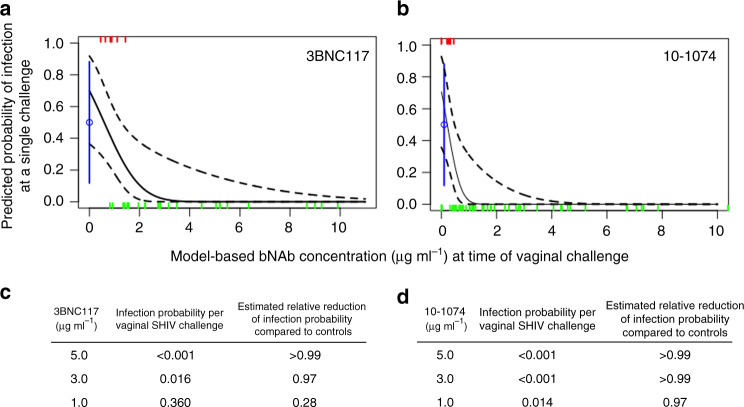
Antibody concentration predicts the probability of infection following vaginal SHIV_AD8-EO_ challenge. Probit regression analysis was used to estimate the per-challenge probability of vaginal SHIV acquisition as a function of imputed 3BNC117 concentrations in plasma among macaques that were administered 3BNC117 singly (**a**) or 10–1074 concentrations in plasma among animals that received 10–1074 in combination with 3BNC117 (**b**). The per-challenge probability of infection among untreated control animals was 0.50 and is denoted in blue. Tick marks represent SHIV challenge events that either did (red) or did not (green) result in systemic infection. The solid black or gray line is the model-based prediction; dashed black lines depict 90% pointwise confidence intervals. Model-based estimates of SHIV infection probabilities following a single vaginal SHIV challenge at the indicated plasma concentrations of 3BNC117 (**c**) or 10–1074 (**d**).

We first used probit regression to estimate the per-challenge probability of SHIV infection as a function of imputed bNAb levels in plasma among animals in the vaginal challenge cohorts (Fig. 4). Among macaques that received 3BNC117 singly, this model estimated infection probabilities of <0.001, 0.016, or 0.36 at 3BNC117 concentrations of 5.0, 3.0, or 1.0 μg ml^−1^, respectively. These estimates represent >100-, 31-, and 1.4-fold reductions in infection probability, respectively, from that of no-antibody controls whose per-challenge infection probability was 0.50 (95% CI: 0.11–0.88). Bootstrap fitting of this model estimated a 0.01 probability of infection following a single vaginal SHIV challenge at a plasma 3BNC117 concentration of 3.22 μg ml^−1^ (95% CI: 1.62, 4.69 μg ml^−1^).

Among macaques in the combination treatment group, probit regression estimated per-challenge infection probabilities of <0.001, <0.001, or 0.014 at 10–1074 concentrations of 5.0, 3.0, or 1.0 μg ml^−1^, respectively. These estimates represent >100-, >100-, and 36-fold reductions as compared with untreated controls. Bootstrap fitting of the model showed a plasma 10–1074 concentration of 1.13 μg ml^−1^ (95% CI: 0.51, 1.53 μg ml^−1^) predicted a 0.01 probability of infection following a single vaginal SHIV_AD8-EO_ challenge. Thus, among vaginally challenged macaques, the estimated plasma bNAb concentration corresponding to a 0.01 probability of infection was three times as high for 3BNC117 [3.22 μg ml^−1^ (95% CI: 1.62, 4.69 μg ml^−1^)] than 10–1074 [1.13 μg ml^−1^ (95% CI: 0.51, 1.53 μg ml^−1^)], which reflects the relatively lower neutralization potency of 3BNC117, as compared with 10–1074, against the challenge virus.

In contrast, comparison of the correlates of 10–1074-mediated protection between the penile and vaginal SHIV challenge models showed no significant difference—the estimated plasma 10–1074 concentration that corresponded to a 0.01 probability of SHIV_SF162P3_ infection via the penis [2.28 μg ml^−1^ (95% CI: 1.27, 3.47 μg ml^−1^)] was not different from that against SHIV_AD8-EO_ infection vaginally [1.13 μg ml^−1^ (95% CI: 0.51, 1.53 μg ml^−1^)].

## Discussion

HIV infection of the penis has contributed substantially to the global HIV epidemic, as a majority of the ~18 million men living with HIV worldwide became infected through heterosexual transmission^46^. However, preclinical animal modeling to assess the efficacy of candidate HIV interventions against penile infection has lagged that targeting rectal or vaginal infection routes. Here, using a recently developed macaque model^33^, we provide a preclinical evaluation of bNAb-mediated protection against penile SHIV infection.

Our results demonstrated durable protection (up to 19 weeks) against penile SHIV_SF162P3_ infection of macaques following a single subcutaneous injection of 10–1074. Probit regression modeling estimated that 10–1074 reduced the per-challenge penile infection risk by >2-logs, from 0.25 among untreated controls to <0.001 among macaques with a plasma 10–1074 concentration of 5.0 μg ml^−1^. In phase-I studies among HIV uninfected people, 10–1074 concentrations > 5.0 μg ml^−1^ were observed for at least 8 weeks following either a single infusion of 10–1074 at 3, 10, or 30 mg kg^−1^ or repeated infusions, once every 8 weeks, of 10–1074 in combination with 3BNC117 at 10 mg kg^−1 17,20^. If directly translatable, our results suggest that relatively infrequent dosing with 10–1074 might provide a high level of protection against penile HIV infection among men.

Our vaginal challenge study also provided information on correlates of bNAb-mediated protection. Among macaques administered 10–1074 and 3BNC117 in combination, protection was attributed to 10–1074 due to the observed difference in antibody pharmacokinetics following subcutaneous administration that resulted in a period of effective 10–1074 monotherapy preceding SHIV_AD8-EO_ infection. Probit regression modeling estimated that per-challenge vaginal infection risk was reduced by >2-logs, from 0.50 among untreated controls to <0.001 among macaques with a plasma 10–1074 concentration of 3.0 μg ml^−1^ or greater.

A limitation in directly comparing the correlates of 10–1074-mediated protection against penile or vaginal infection in this study is that the underlying animal models utilized different challenge virus strains and doses. Here, our penile and vaginal challenge models used SHIVs that are similarly susceptible to neutralization by 10–1074, with IC_80_ values of 0.20 or 0.25 μg ml^−1^ against the replication competent challenge virus stock of SHIV_SF162P3_ or SHIV_AD8-EO_, respectively. Regarding challenge virus dose selection, model feasibility necessitates that each SHIV exposure presents a relatively higher risk of infection to macaques, than does exposure of men or women to an HIV-infected source, for which the per-exposure infection rates are low (insertive vaginal—0.0004, insertive rectal—0.0011, receptive vaginal—0.0008)^1^. For these macaque studies, challenge virus doses were selected empirically to normalize the number of challenges required to infect animals via penile or vaginal routes and resulted in a median of 2 or 2.5 challenges, respectively. Importantly, the bootstrapped estimate of the per-challenge probability of infection among untreated control animals in the penile SHIV_SF162P3_ model [0.25 (95% CI: 0.13–0.41)] was not significantly different from that among vaginal SHIV_AD8-EO_ controls [0.50 (95% CI: 0.11–0.88)]. Thus, macaques were challenged with SHIVs, via a penile or vaginal route, under model conditions of comparable stringency. These model-specific differences notwithstanding, we found no significant difference between the plasma 10–1074 concentration that reduced per-exposure infection risk to 0.01 against penile SHIV_SF162P3_ or vaginal SHIV_AD8-EO_ challenges, and risk reduction was greater than 100-fold for both groups when plasma 10–1074 concentrations were 5.0 μg ml^−1^ or higher.

Likewise, these correlates of protection against penile or vaginal infection compare favorably with those reported for 10–1074-mediated protection against repeated rectal SHIV_AD8-EO_ challenges among macaques^23^. For example, at a plasma 10–1074 concentration of 1.0 μg ml^−1^, 63 or 84% reductions in per-challenge infection risk were observed among macaques challenged repeatedly with SHIV_SF162P3_ via the penis or SHIV_AD8-EO_ rectally^23^. These findings are consistent with an earlier study showing passively administered bNAb PGT126 protected similarly against a single high-dose challenge with SHIV_SF162P3_ via rectal or vaginal routes^47^.

The site(s) and mechanism(s) by which passively administered bNAbs confer protection against penile or other mucosal routes of infection are not known fully. Transudation or active (FcRn-mediated) transport of bNAbs from circulation to mucosal tissues or secretions would position these effector molecules at the point of virus entry, where their neutralization or Fc-related antiviral functions may be expected to have greatest impact to prevent either initial infection or local amplification and systemic spread of a nascent infection^48–51^. Observation that the plasma correlates of 10–1074-mediated protection were not different against penile or vaginal infection may reflect similar bNAb pharmacokinetics among these anatomic compartments or may reflect common antiviral effects occurring elsewhere than the mucosal portal of virus entry. Thus, a second limitation of this study was the lack of mucosal sampling to determine bNAb pharmacokinetics in penile tissues or secretions. However, because our primary study objective was protective efficacy, we chose not to collect penile samples from animals being challenged, out of concern that the collection procedures themselves would alter susceptibility to SHIV acquisition. Future studies to characterize the pharmacokinetics of bNAbs in penile tissues or secretions, following passive administration, should be informative in this regard.

We note that our penile infection model is a model of HIV infection among uncircumcised men. Because 60–70% of adult men globally are not circumcised^52,53^, this model has broad public health relevance. However, penile tissues other than the foreskin are relevant for HIV infection, as evidenced by the incomplete protection that circumcision confers against penile infection^54–56^, as well as the wide distribution of HIV target cells among other penile tissues, including the urethra and glans^25,57,58^. Although our current challenge model simultaneously exposes multiple penile tissues to SHIV, it is of interest to determine whether removal of the foreskin via circumcision would alter the capacity of bNAbs to protect remaining penile tissues from infection. A final model limitation is that the nontraumatic technique used for penile challenges with SHIV does not account for any trauma that might occur to the penis during sexual intercourse.

In summary, we have utilized a recently developed macaque model of penile HIV infection to demonstrate protective efficacy of a passively administered bNAb against this route of infection. We determined plasma 10–1074 concentrations as correlates of protection against penile or vaginal SHIV acquisition and compared these to values reported to protect against rectal SHIV infection. Despite model-specific differences, the findings from these macaque studies show that large reductions in infection probability for all major routes of HIV acquisition could be demonstrated at clinically relevant levels of circulating bNAbs. Such an overall indication from macaque modeling should facilitate dose selection of bNAbs for clinical advancement of immunoprophylaxis against HIV.

## Methods

### Macaques

Thirty adult rhesus macaques (*Macaca mulatta*) were used to perform the penile or vaginal SHIV challenge studies, respectively. All animals were housed at the Centers for Disease Control and Prevention (CDC; Atlanta, GA) in accordance with the *Guide for the Care and Use of Laboratory Animals* (8th edition) in an AAALAC-accredited facility, according to institutional standard operating procedures. For housing, macaques were maintained in cages that met or exceeded the minimum size requirements as stipulated in the Guide. Animals were provided enrichments that included objects to manipulate, assortments of fresh fruits and vegetables, suitable feeding methods (foraging and task-oriented), and humane interactions with caregivers and research staff. Prior to the initiation of virus challenges, compatible macaques were pair-housed to the extent possible. Animal studies were approved by the CDC Institutional Animal Care and Use Committee (IACUC, protocol 2804GARMONC). To minimize animal discomfort or suffering, all biomedical procedures were performed on animals under ketamine (10 mg kg^−1^) or Telazol (2–6 mg kg^−1^) anesthesia.

### Challenge virus stocks

Preparation and characterization of the cell-free SHIV_SF162P3_ stock used to perform penile challenges has been described^33^—the undiluted stock had a titer of 2430 TCID_50_ ml^−1^, determined on whole, unstimulated primary rhesus PBMCs. A cell-free stock of SHIV_AD8-EO_, which was used to perform vaginal challenges, was prepared as follows. Infectious virus was obtained in supernatants of 293T cell cultures at 48 h following FuGene 6 (Promega)-mediated transfection of plasmid pSHIV AD8-EO (kindly provided by Malcolm Martin, NIAID). Virus in 293T culture supernatants was amplified in rhesus macaque PBMCs following in vitro depletion of CD8^+^ cells (Dynabeads CD8, ThermoFisher) and stimulation with Concanavalin-A (Sigma-Aldrich). Supernatants were clarified via centrifugation, aliquoted and stored in the vapor phase of liquid nitrogen. The undiluted SHIV_AD8-EO_ stock had a titer of 3600 TCID_50_ ml^−1^, determined on whole, unstimulated primary rhesus PBMCs.

### Antibodies and passive immunization

Monoclonal antibodies 10–1074 and 3BNC117 were produced in the laboratory of M.C.N. and formulated individually for injection at concentrations ranging between 49.5 and 53 mg ml^−1^ in 5 mM acetate, 280 mM trehalose, 0.05% Tween20 (pH 5.2) or 10 mM l-histidine, 280 mM trehalose, 0.05% Tween20 (pH 5.5), for 10–1074 and 3BNC117, respectively^17,18^. Antibodies were administered via subcutaneous injection in macaques at 10 mg kg^−1^ in the upper back (3BNC117 on the left side, 10–1074 on the right side) via 22G1 needle; injection volumes were <2 ml per injection site.

### Virus challenges

SHIV challenges were performed once weekly, via penile or vaginal routes, until systemic infection was confirmed by detection of vRNA in plasma. Penile SHIV challenges were performed by administering 200 TCID_50_ into the prepuce (foreskin) pouch and 16 TCID_50_ virus into the distal urethra. Urethral inoculations were performed using a ‘no-contact’ technique in which the lobes of the glans were manually flared to expose the navicular fossa and a 20 μl inoculum volume was expelled from a micropipettor tip positioned 1–5 mm above the center of the exposed urethral opening^33^. This technique avoids all contact of the pipet tip with urethral tissue and precludes inadvertent abrasion of the urethral epithelium. SHIV_SF162P3_ was used for penile challenges as it is sensitive to neutralization by bNAb 10–1074, and the penile model had been optimized with SHIV_SF162P3_. For vaginal SHIV challenge, macaques were nontraumatically administered 300 TCID_50_ virus, in a 1 ml volume, via an inserted pediatric nasogastric feeding tube of adjusted length. SHIV_AD8-EO_ was selected for vaginal studies because we wanted to evaluate two bNAbs—3BNC117 and 10–1074, both of which neutralize this virus.

### Viral load assay

Viral RNA in plasma was quantified using a real-time reverse transcription PCR assay to detect the SIV *gag* gene^59^. SHIV virions were concentrated from 1 ml plasma samples via ultracentrifugation (100,000 g for 30 min at 4 °C) and subjected to RNA extraction using NucliSens reagents (Biomerieux). One-step RT-PCR was performed using the SuperScript III Platinum One-Step qRT-PCR Kit with ROX (ThermoFisher) with forward primer (SIVp1f1) 5′-GCCAACAGGCTCAGAAAATTTAA-3′, reverse primer (SIVp1r1) 5′-TCCTCAGTGTGTTTCACTTTCTCTTC-3′ and probe 5′-HEX-AGCCTTTATAATACTGTCTGCGTCATCTGGTGC-BHQ1-3′. The limit of detection was 60 viral RNA copies per ml.

### Determination of 10–1074 and 3BNC117 concentrations

Concentrations of 10–1074 and 3BNC117 in plasma were determined using TZM-bl neutralization assays using pseudovirus X2088.c9, which is sensitive to neutralization by 10–1074 but resistant to 3BNC117, or Q769.d22, which is sensitive to 3BNC117, but resistant to 10–1074^20^. Inhibitory dilution 50% (ID_50_) titers of pseudovirus neutralization by plasma samples were determined via five-parameter curve fitting. Plasma antibody concentration was calculated by multiplying a sample’s ID_50_ titer by the inhibitory concentration 50% value from 10–1074 or 3BNC117 reference lots, which were assayed in parallel. Plasma samples were measured against murine leukemia virus to detect any nonspecific activity. All samples were assayed in duplicate. Induction of endogenous (rhesus) antibody responses against SHIV Env following repeated SHIV challenges, but prior to systemic infection, could confound use of the TZM-bl assay to determine plasma bNAb concentrations. SHIV_SF162P3_ Env-specific antibody responses were not detected prior to SHIV breakthrough among macaques that received 10–1074 and were challenged repeatedly with SHIV_SF162P3_ via the penis (Supplementary Fig. 4).

### Detection of antidrug antibody (ADA) responses

ADA responses were evaluated in macaque plasma samples via ELISA. ELISA plates (96-well, high binding plates (Corning)) were coated overnight with 1 μg 10–1074 per well, then blocked with phosphate buffered saline containing 1% bovine serum albumin (PBS-1%BSA). Plasma samples were diluted 1:50 in PBS-1%BSA, added to the plates (100 μl per well) and incubated for 1.5–2.0 h at 37 °C. Plates were washed and incubated with mouse antirhesus IgG heavy chain-horseradish peroxidase (HRP) preadsorbed (Abcam) for 30 min at 37 °C. Plates were washed and assayed for HRP activity by the addition of tetramethylbenzidine (1-Step Ultra TMB, ThermoFisher). Color development was arrested via the addition of sulfuric acid to a final concentration of 1 N and absorbance at 450 nm was measured using a BioTek ELx808 plate reader.

### Statistical analysis

Statistical comparisons between groups using *t*-, Mann–Whitney, or log-rank tests were conducted using Prism 7.0 (GraphPad Software, Inc., San Diego, CA). Probit modeling and bootstrap analyses were conducted in R version 3.4.4.

### Reporting summary

Further information on research design is available in the Nature Research Reporting Summary linked to this article.

## Supplementary information

Supplementary Information

Reporting Summary

## Data Availability

All relevant data are available from the authors. The source data underlying Figs. 1e, 3f, and 3g are included in Supplementary Tables 1, 2, and 3, respectively. The source data underlying Figs. 1b, c, f, 3b, c, d, h are provided as a Source Data File.

## Source data

Source Data
