# Supplementary material for: Durable protection against repeated penile exposures to simian-human immunodeficiency virus by broadly neutralizing antibodies
Source: Nat Commun. 2020 Jun 24;11:3195. doi: 10.1038/s41467-020-16928-9 (PMC7314794; doi:10.1038/s41467-020-16928-9)
Supplement: Supplementary file 1 — Supplementary Information [file 41467_2020_16928_MOESM1_ESM.pdf]

Supplementary Information

Durable protection against repeated penile exposures to simian-human immunodeficiency virus by broadly neutralizing antibodies

Garber et al.

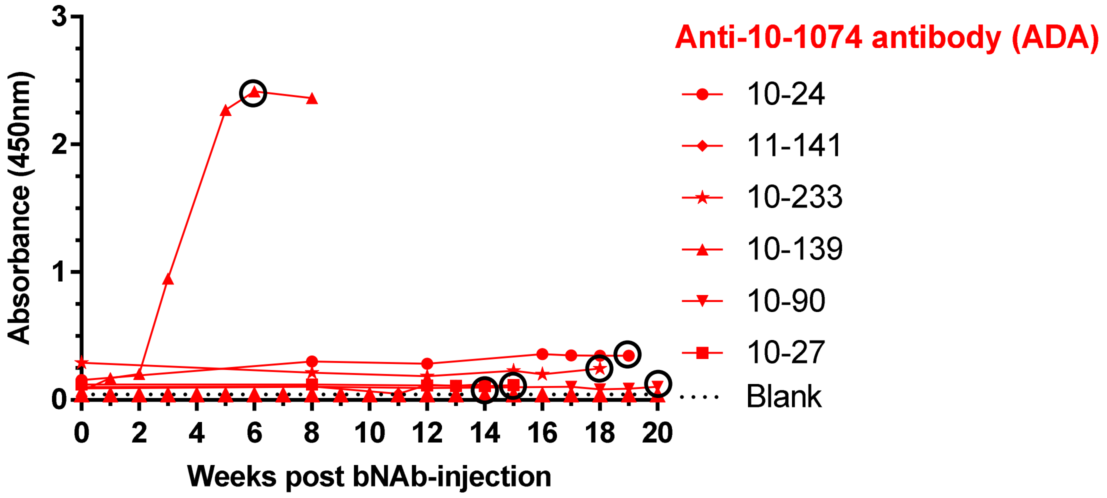

Supplementary Figure 1. Development of anti-10-1074 antibody responses (ADA) among macaques administered 10-1074 and challenged with SHIV via the penis. Longitudinal plasma samples were assayed via ELISA for 10-1074-specific IgG responses. Open circles denote the timepoint of SHIV breakthrough for each animal.

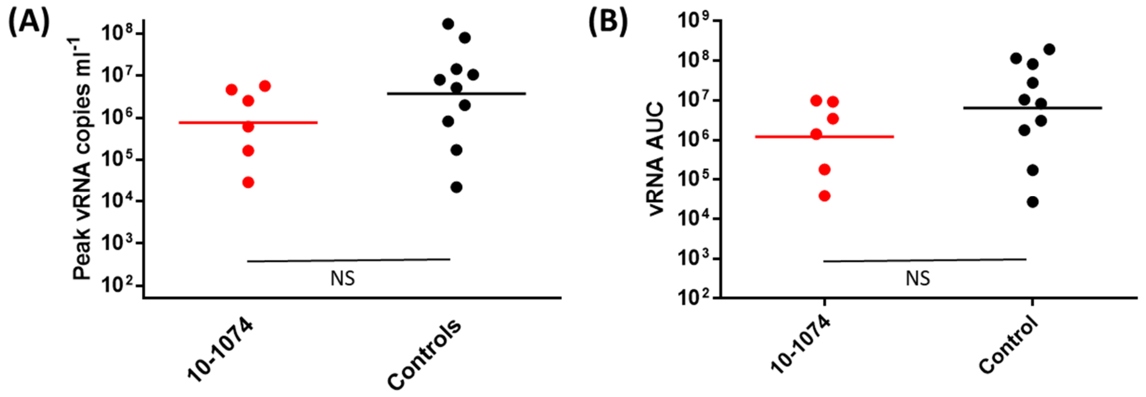

Supplementary Figure 2. Plasma viremia following penile SHIV$_{SF162P3}$ infection

(**A**) Peak plasma viremia or **(B)** vRNA area under the curve through 7 weeks post-infection following penile SHIV infection among 10-1074-treated macaques or untreated controls. At each timepoint for each macaque, vRNA values were determined as the average of duplicate samples. . Animal group sizes were 6 (10-1074) or 10 (Control), as shown. Lines denote geometric means for each group. NS = not significant (Mann-Whitney test, two-sided).

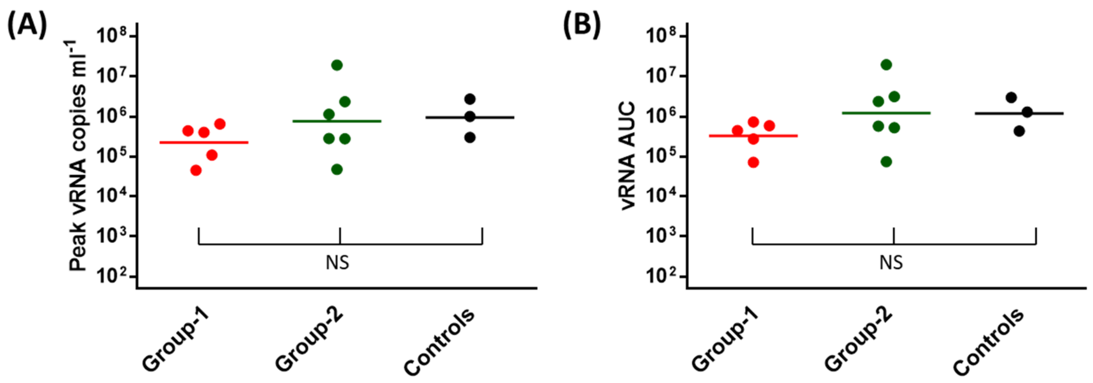

Supplementary Figure 3. Plasma viremia following vaginal $SHIV_{AD8\text{-}EO}$ infection

(A) Peak plasma viremia or **(B)** vRNA area under the curve through 7 weeks post-infection, following vaginal SHIV acquisition among animals administered a combination of 10-1074 and 3BNC117 (Group-1, N=5)), 3BNC117 only (Group-2, N=6)) or untreated controls (N=3). At each timepoint for each macaque, vRNA values were determined as the average of duplicate samples. Lines denote geometric means for each group. NS = not significant (Kruskal-Wallis test, two-sided).

A.

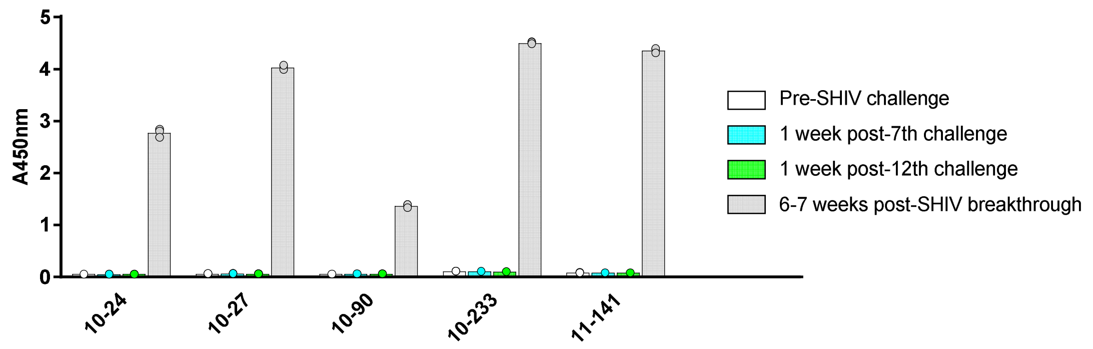

B.

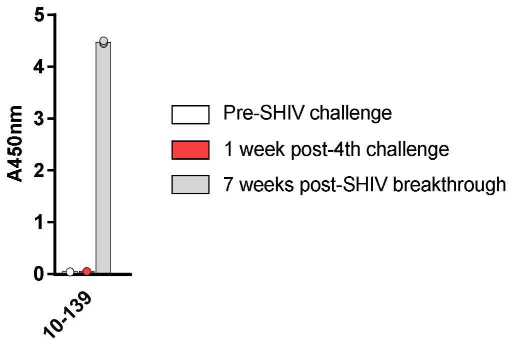

Supplementary Figure 4. Rhesus antibody responses against $SHIV_{SF162P3}$ Env following repeated penile $SHIV_{SF162P3}$ challenges

Rhesus $SHIV_{SF162P3}$ Env-specific antibody responses were evaluated in plasma samples via ELISA among macaques administered 10-1074, challenged repeatedly with $SHIV_{SF162P3}$ via the penis, who exhibited SHIV breakthrough following 13-19 weekly challenges (A) or 5 weekly challenges (B). ELISA plates (96-well, high binding plates (Corning)) were coated overnight with 1µg $SHIV_{SF162P3}$ Env (gp120) (Immune Technology Corp.) per well, then blocked with phosphate buffered saline containing 1% bovine serum albumin (PBS-1%BSA). Plasma samples were diluted 1:50 in PBS-1%BSA, added to the plates (100µl per well) and

incubated for 1.5 hour at 37ºC. Plates were washed and incubated with mouse anti-rhesus IgG heavy chain-horseradish peroxidase (HRP) preadsorbed (Abcam) for 30 minutes at 37ºC. Plates were washed and assayed for HRP activity by the addition of tetramethylbenzidine (1-Step Ultra TMB, ThermoFisher). Color development was arrested via the addition of sulfuric acid to a final concentration of 1N and absorbance at 450nm was measured using a BioTek ELx808 plate reader. Plasma samples were assayed in triplicate - individual values (circles) and mean values (bars) are shown. Panels (A) and (B) show representative results from one of two independent experiments.

Supplementary Table 1. 10-1074 antibody concentration (μg ml^{-1}) in macaque plasma following subcutaneous injection at week (-1)

10-1074

Week	10-24	10-27	10-90	10-139	10-233	11-141
-1	<0.10	<0.10	<0.10	<0.10	<0.10	<0.10
0	40.90	24.60	27.20	27.50	35.50	60.00
2	24.30	14.20	16.40	24.70	23.00	18.80
4	12.40	6.40	10.00	<0.10	13.10	9.50
5				<0.10		
6	6.10	3.40	6.20		11.00	4.20
8	3.40	2.10	2.60		3.60	3.20
10	3.20	1.00	1.70		3.40	1.70
12	1.20	0.70	0.80		2.20	0.70
13		0.60				0.70
14	0.80	0.70	0.40		1.10	
16	0.50		0.40		0.60	
17	0.30				0.60	
18	0.40		0.20			
19			0.20			

Supplementary Table 2. bNAb concentrations (μg ml^{-1}) in plasma of macaques co-administered 3BNC117 and 10-1074 via subcutaneous injections at week (-1)

3BNC117

Week	RQ-02E035	RQ-02E060	RQ-8433	RQ-8684	RQ-8918
-1	<0.24	<0.24	<0.24	<0.24	<0.24
0	5.20	6.00	2.00	8.50	7.90
1	3.60	4.20	1.00	5.60	4.30
2	2.90	2.20	0.60	3.30	3.40
3	1.90	1.40	<0.24	4.20	1.60
4	1.10	0.60	<0.24	1.80	1.20
5	0.70	0.50	<0.24	0.60	0.50
6	0.80	<0.24	<0.24	0.50	0.30
7	0.30	<0.24	<0.24	0.40	<0.24
8	<0.24	<0.24	<0.24	<0.24	<0.24
9	<0.24	<0.24	<0.24	<0.24	<0.24

10-1074

Week	RQ-02E035	RQ-02E060	RQ-8433	RQ-8684	RQ-8918
-1	<0.07	<0.07	<0.07	<0.07	<0.07
0	51.10	35.10	26.10	36.60	29.60
1	24.40	17.20	15.30	25.90	13.70
2	12.80	9.80	9.50	13.60	12.00
3	12.40	5.90	6.20	11.00	7.10
4	7.30	3.60	5.50	9.60	4.60
5	4.20	2.80	4.10	4.90	2.20
6	3.00	1.80	2.30	4.10	1.70
7	2.10	1.40	1.40	3.60	1.00
8	1.60	0.80	0.90	1.70	0.80
9	1.00	0.60	0.60	1.40	0.40
10	0.50	0.20	0.20	0.50	0.10
11	0.30	0.10	0.10	0.30	<0.07
12	0.20	<0.07	0.10	0.30	<0.07
13	0.10	<0.07	<0.07	0.20	<0.07
14	0.10	<0.07	<0.07	<0.07	<0.07
15	<0.07	<0.07	<0.07	<0.07	<0.07
16	<0.07	<0.07	<0.07	<0.07	<0.07
17	<0.07	<0.07	<0.07	<0.07	<0.07
18	<0.07	<0.07	<0.07	<0.07	<0.07

Supplementary Table 3. 3BNC117 antibody concentration ($\mu g\ ml^{-1}$) in plasma of macaques administered 3BNC117 singly via subcutaneous injection at week (-1)

3BNC117

Week	07-155	RQ-8011	RQ-8428	RQ-8944	RQ-9200	RQ-9207
-1	<0.24	<0.24	<0.24	<0.24	<0.24	<0.24
0	4.90	8.30	8.70	1.70	7.70	10.50
1	2.70	5.20	5.40	1.60	6.90	4.50
2	1.80	3.70	3.60	0.90	5.90	2.90
3	0.90	1.60	2.40	0.70	2.60	1.30
4	0.40	0.60	1.00	0.40	2.10	0.80
5	<0.24	<0.24	1.10	0.30	1.80	0.50
6	<0.24	<0.24	0.70	<0.24	1.00	<0.24
7	<0.24	<0.24	0.30	<0.24	0.80	<0.24
8	<0.24	<0.24	<0.24	<0.24	<0.24	<0.24
9	<0.24	<0.24	<0.24	<0.24	<0.24	<0.24
