# Supplementary material for: Durable protection against repeated penile exposures to simian-human immunodeficiency virus by broadly neutralizing antibodies
Source: Nat Commun. 2020 Jun 24;11:3195. doi: 10.1038/s41467-020-16928-9 (PMC7314794; doi:10.1038/s41467-020-16928-9)
Supplement: Supplementary file 2 — Reporting Summary [file 41467_2020_16928_MOESM2_ESM.pdf]

nature research

Corresponding author(s): David A. Garber

Last updated by author(s): 05/29/2020

# Reporting Summary

Nature Research wishes to improve the reproducibility of the work that we publish. This form provides structure for consistency and transparency in reporting. For further information on Nature Research policies, see Authors & Referees and the Editorial Policy Checklist.

## Statistics

For all statistical analyses, confirm that the following items are present in the figure legend, table legend, main text, or Methods section.

| n/a | Confirmed | |
|---|---|---|
| ☐ | ☒ | The exact sample size (*n*) for each experimental group/condition, given as a discrete number and unit of measurement |
| ☐ | ☒ | A statement on whether measurements were taken from distinct samples or whether the same sample was measured repeatedly |
| ☐ | ☒ | The statistical test(s) used AND whether they are one- or two-sided<br>*Only common tests should be described solely by name; describe more complex techniques in the Methods section.* |
| ☒ | ☐ | A description of all covariates tested |
| ☒ | ☐ | A description of any assumptions or corrections, such as tests of normality and adjustment for multiple comparisons |
| ☐ | ☒ | A full description of the statistical parameters including central tendency (e.g. means) or other basic estimates (e.g. regression coefficient) AND variation (e.g. standard deviation) or associated estimates of uncertainty (e.g. confidence intervals) |
| ☐ | ☒ | For null hypothesis testing, the test statistic (e.g. *F*, *t*, *r*) with confidence intervals, effect sizes, degrees of freedom and *P* value noted<br>*Give P values as exact values whenever suitable.* |
| ☒ | ☐ | For Bayesian analysis, information on the choice of priors and Markov chain Monte Carlo settings |
| ☒ | ☐ | For hierarchical and complex designs, identification of the appropriate level for tests and full reporting of outcomes |
| ☐ | ☒ | Estimates of effect sizes (e.g. Cohen's *d*, Pearson's *r*), indicating how they were calculated |

*Our web collection on statistics for biologists contains articles on many of the points above.*

## Software and code

Policy information about availability of computer code

| | |
|---|---|
| Data collection | No software was used for data collection. |
| Data analysis | Statistical comparisons between groups using t-, Mann-Whitney- or log-rank tests were conducted using Prism 7.0 (GraphPad Software, Inc, San Diego, CA). Probit modeling and bootstrap analyses were conducted in R version 3.4.4. |

For manuscripts utilizing custom algorithms or software that are central to the research but not yet described in published literature, software must be made available to editors/reviewers. We strongly encourage code deposition in a community repository (e.g. GitHub). See the Nature Research guidelines for submitting code & software for further information.

## Data

Policy information about availability of data

All manuscripts must include a data availability statement. This statement should provide the following information, where applicable:

- Accession codes, unique identifiers, or web links for publicly available datasets
- A list of figures that have associated raw data
- A description of any restrictions on data availability

All data generated or analysed during the study are included in the article and its supplementary information or source data files.

# Field-specific reporting

Please select the one below that is the best fit for your research. If you are not sure, read the appropriate sections before making your selection.

☒ Life sciences ☐ Behavioural & social sciences ☐ Ecological, evolutionary & environmental sciences

For a reference copy of the document with all sections, see nature.com/documents/nr-reporting-summary-flat.pdf

# Life sciences study design

All studies must disclose on these points even when the disclosure is negative.

| | |
|---|---|
| Sample size | A total of 30 rhesus macaques (Macaca mulatta) was used to perform the penile and vaginal challenge studies. Ten control animals, which were challenged with SHIV162P3 via the penile route in the absence of any bNAb infusion, were reported previously in a manuscript describing development of the penile SHIV challenge model (Garber DA, et al. PLoS One 13, e0194837 (2018)). Group sizes of up to 6 macaques, among groups of animals that were administered bNAb(s), is consistent with other published studies utilizing repeated limiting-dose virus challenge models. |
| Data exclusions | No data were excluded. |
| Replication | All samples were assayed in duplicate for vRNA or TZM-bl neutralization assays. |
| Randomization | Animals were allocated by gender into study arms as required by the study design (penile vs vaginal challenge). Within each arm, animals were assigned randomly to treatment or control groups. |
| Blinding | Blinding was not relevant in this study - animal samples were analysed using objective, standardized assays that included appropriate controls. |

# Reporting for specific materials, systems and methods

We require information from authors about some types of materials, experimental systems and methods used in many studies. Here, indicate whether each material, system or method listed is relevant to your study. If you are not sure if a list item applies to your research, read the appropriate section before selecting a response.

## Materials & experimental systems

| n/a | Involved in the study | |
|---|---|---|
| ☐ | ☒ | Antibodies |
| ☐ | ☒ | Eukaryotic cell lines |
| ☒ | ☐ | Palaeontology |
| ☐ | ☒ | Animals and other organisms |
| ☒ | ☐ | Human research participants |
| ☒ | ☐ | Clinical data |

## Methods

| n/a | Involved in the study | |
|---|---|---|
| ☒ | ☐ | ChIP-seq |
| ☒ | ☐ | Flow cytometry |
| ☒ | ☐ | MRI-based neuroimaging |

## Antibodies

| | |
|---|---|
| Antibodies used | 10-1074 and 3BNC117 were generated by the Nussenzweig laboratory |
| Validation | Neutralization activities of bNAbs against challenge viruses were determined using TZM-bl cell assay. |

## Eukaryotic cell lines

Policy information about cell lines

| | |
|---|---|
| Cell line source(s) | TZM-bl: NIH AIDS Reagent Program |
| Authentication | Not authenticated |
| Mycoplasma contamination | Not tested |
| Commonly misidentified lines (See ICLAC register) | No commonly misidentified cell lines were used in the study. |

## Animals and other organisms

Policy information about studies involving animals; ARRIVE guidelines recommended for reporting animal research

| | |
|---|---|
| Laboratory animals | Rhesus macaques (Macaca mulatta) or Indian- or Chinese-genetic origin, as indicated; male or female; 4.8-10.7 years old |
| Wild animals | The study did not involve wild animals. |
| Field-collected samples | The study did not involve samples collected from the field. |

Ethics oversight

The study was approved by the Institutional Animal Care and Use Committee of the Centers for Disease Control and Prevention (protocol 2804GARMONC) and was performed in compliance with the Public Health Service Policy on Humane Care and Use of Laboratory Animals, the Animal Welfare Act and the Guide for the Care and Use of Laboratory Animals.

Note that full information on the approval of the study protocol must also be provided in the manuscript.
